# Efficacy of Ferrous Sulphate in the Management of Iron Deficiency Anemia in Children

**DOI:** 10.7759/cureus.84826

**Published:** 2025-05-26

**Authors:** Karishma Siraj, Faryal Naz, Shabnam Mahsood, Haseena Ali, Fatima Shafiq, Seema Nawaz, Khadija Manzar, Aizaz Ur Rahman, Sana Siraj, Tahir Hassan

**Affiliations:** 1 Pediatrics, Lady Reading Hospital, Peshawar, PAK; 2 Internal Medicine, South West Medical Hospital, Kohat, PAK; 3 Pediatrics, Hayatabad Medical Complex, Peshawar, PAK; 4 Internal Medicine, Bacha Khan Medical Complex, Mardan, PAK; 5 Orthopedics and Traumatology, Hayatabad Medical Complex, Peshawar, PAK

**Keywords:** efficacy, ferrous sulfate supplements, hemoglobin: hb, internal medicine & pediatrics, iron deficiency anemia (ida), serum ferritin

## Abstract

Background

Iron deficiency anemia (IDA) is a highly prevalent disorder among the pediatric population and has a significant impact on growth and health. Ferrous sulfate is commonly prescribed for the treatment of IDA; however, its efficacy in the pediatric population requires further research.

Objective

This study aimed to evaluate the efficacy of ferrous sulfate in improving hemoglobin (Hb) and ferritin levels in children diagnosed with IDA.

Methods

A quasi-experimental study was conducted involving 108 participants aged 1-8 years who were diagnosed with IDA. Participants received ferrous sulfate supplements for three months. Hemoglobin and serum ferritin levels were measured both at baseline and after three months of treatment.

Results

Ferrous sulfate administration resulted in a significant increase in hemoglobin levels by an average of 3.11 mg/dl and a net increase in ferritin levels by 5.9 ng/ml after 3 months of treatment. Additionally, a positive association was found between low Hb levels and underweight children*.*

Conclusion

Ferrous sulfate is an effective therapeutic agent for managing IDA in children, leading to significant improvements in hemoglobin and ferritin levels. Further research involving RCTs with longer follow-up periods is recommended.

## Introduction

Children with iron deficiency anemia (IDA) exhibit symptoms such as paleness, irritability, loss of appetite, and lack of energy. The most commonly utilized preparations are ferrous sulphate and iron polymaltose in its ferric form [1, 2]. The effectiveness, absorption rate, adverse reactions, and price of different formulations differ. Anemia is characterized by hemoglobin levels below the 5th percentile for a specific age group. Anemia is a prevalent global health issue, with children under the age of 5 years being the most vulnerable population [3]. 

The primary cause of anemia in children under two years of age is nutritional inadequacy, which arises from the rapid developmental rate and increased requirement for iron, Vitamin B12, and folic acid. Based on a study conducted in Karachi, anemia is the predominant nutritional deficiency observed in malnourished children in Pakistan, with a prevalence rate of 78% [4]. Approximately 1.62 billion individuals globally suffer from iron-deficient anemia. The incidence of preschool children affected is 47% of the overall population affected [5]. Children aged 6 months to 2 years are more susceptible to developing iron deficiency anemia, with the primary cause being the delayed introduction of solid foods. Nevertheless, the severity of anemia is also linked to recurring bouts of upper respiratory tract infections, diarrhea, trauma, and surgery. Once the root cause of iron deficiency anemia in children has been identified, it is advisable to administer iron replacement therapy to restore normal hemoglobin levels [6].

The most commonly utilized preparations are ferrous sulfate and iron polymaltose in its ferric form. The effectiveness, absorption rate, adverse reactions, and price of different formulations differ. One study evaluated the effectiveness of ferrous sulfate [7]. The findings demonstrate that ferrous sulfate is the preferred treatment for iron-deficient anemia because of its high efficacy, affordability, and improved tolerability. Treatment failure often occurs as a result of non-compliance with medicine, unpleasant taste, and the absence of clear data-based guidelines. [8]. A study reported that ferrous sulfate was (86.7%) effective among children in the management of iron deficiency anemia [9].

Iron deficiency anemia is a widespread global health problem, especially affecting children, and has significant implications for growth, cognitive development, and general welfare. The Global Burden of Disease study showed that dietary iron deficiency remains a major health concern with a global prevalence of 16.7%, particularly affecting female individuals, children, and residents in low-socio-demographic index (SDI) countries [10]. Due to the paucity of literature on this subject locally, this study aimed to determine the efficacy of ferrous sulfate in the management of iron deficiency anemia in children at our hospital. The results of this study will assist healthcare providers in understanding the efficacy and tolerability of this drug, which is essential for making informed decisions regarding the optimal iron supplementation strategy for each patient to achieve promising results.

## Materials and methods

Study design and setting

This study employed a quasi-experimental design conducted in the pediatrics department of Lady Reading Hospital-MTI, Peshawar, Pakistan, from June 7, 2024, to April 6, 2025. The study protocol was approved by the Institutional Review Board, Lady Reading Hospital, MTI, Peshawar (approval no. Ref No. 62/LRH/MTI) and the competent authority of the hospital following national regulations. The parents or guardians of all patients provided written informed consent at enrollment after being informed of the methods, risks, and potential benefits of the study.

Inclusion criteria

Both male and female children aged 1 to 8 years with a diagnosis of iron deficiency anemia, defined by blood hemoglobin (Hb) levels lower than 10.5 g/dL and serum ferritin concentrations of less than 15 ng/ mL, were eligible.

Exclusion criteria

Children were excluded if they presented with anemia due to causes other than iron deficiency (ID), including inflammatory anemia, anemia from marrow failure, hemoglobinopathies (such as sickle-cell disease and thalassemia), hemolytic anemia, anemia due to acute hemorrhage, chronic renal failure-related anemia, hemochromatosis, secondary iron overload (including from blood transfusions), gastro-duodenal ulcer, inflammatory bowel disease, any digestive disease that could affect iron absorption, or pica. Additionally, children were excluded if they had received oral or parenteral iron treatment within the three weeks leading up to the screening visit, had a history of hypersensitivity to any component of the tested treatment or intolerance to oral iron derivatives, or required long-term treatment known to affect iron absorption. Children who were lost to follow-up were also excluded.

A total of 132 participants who met the study's inclusion criteria were included. Demographic data, such as age, gender, mother's education status, and socioeconomic status, were recorded. All participants with iron deficiency anemia received ferrous sulfate, administered at a dosage of 6 mg/kg/day in syrup form for 90 days (three months). Efficacy was defined as an improvement in hemoglobin and serum ferritin levels after 90 days of treatment. The hemoglobin level was set at greater than 10.5 mg/dL, and the serum ferritin level at greater than 15 ng/ml. The entire evaluation was conducted under the supervision of an experienced consultant with at least 5 years of post-fellowship experience. A pre-designated structured questionnaire was used to record each patient's details (Appendices).

Sample size

 A consecutive non-probability sampling technique was used, and the sample size was calculated using the WHO sample size calculator using the following parameters: efficacy of ferrous sulfate (86.7%) among children in managing iron deficiency anemia, confidence level of 95%, and margin of error of 5% [9]. The sample size was determined to be 178. However, for convenience, we included 132 participants. Some children were lost to follow-up, and others had poor drug compliance, which led to their exclusion. Ultimately, 108 children were followed up for three months. 

Statistical analysis 

We analyzed our data using IBM SPSS v.27 (IBM Corp., Armonk, USA) and Jamovi v.2.6.44 (www.jamovi.org). Mean + SD or Median (IQR) was calculated for numerical data, such as age, hemoglobin level, and serum ferritin level. The Shapiro-Wilk test was used to check the normality of the data. Frequencies and percentages were calculated for categorical data such as gender, efficacy, mother's education status, and socioeconomic status. Statistical tests were applied using a p-value < 0.05 as statistically significant. 

## Results

The total number of patients was 108. The mean age was 3.98 ± 2.28 years ( range 1-8 years), and 44 (40.7.6%) and 64 (59.3%) patients were male and female, respectively. The Shapiro-Wilk test showed that the age data were not normally distributed (W = 0.906, p < 0.001). The pre-treatment baseline serum ferritin level was 11.8 ± 1.89 ng/mL (ranging from 8 to 15 ng/mL), and the mean serum hemoglobin level was 8.39 ± 1.21 gm/dL (ranging from 5 to 10.1 gm/dL). After three months of treatment, the mean hemoglobin (HB) level was 11.5 ± 1.45 gm/dL (range 8.5 to 15 gm/dL), indicating a net increase of 3.11 g/dL compared to pretreatment levels. The mean post-treatment ferritin was 17.7 ± 3.34 ng/mL (range 12 to 28 ng/mL) ng/ml, reflecting a net increase of 5.9 ng/mL compared to pretreatment levels. Demographics and patient characteristics with corresponding results are mentioned in Table 1.

**Table 1:** Demographics, Characteristics and Corresponding results Efficacy 'Yes' = hemoglobin greater than 10.5 gm/dL and serum ferritin greater than 15 ng/mL after three months of treatment; Efficacy 'No' = hemoglobin less than 10.5 gm/dL and serum ferritin less than 15 ng/mL after three months of treatment.

Demographics and patient characteristics	Value (N=108)
Gender, n (%)	
male	44 (40.7%)
female	64 (59.3%)
Age, years	
Mean (SD)	4.0 (2.3)
Range	1.0 - 8.0
Weight, kg	
Mean (SD)	13.6 (5.0)
Range	5.9 - 30.0
Pre-treatment Hb, gm/dL	
Mean (SD)	8.4 (1.2)
Range	5.0 - 10.1
Post-treatment Hb, gm/dL	
Mean (SD)	11.5 (1.5)
Range	8.5 - 15.0
Pre-treatment (baseline) ferritin, ng/mL	
Mean (SD)	11.8 (1.9)
Range	8.0 - 15.0
Post-treatment ferritin, ng/mL	
Mean (SD)	17.7 (3.3)
Range	12.0 - 28.1
Nutritional status (centile chart weight for age), n (%)	
Normal	39 (36.1%)
Underweight	68 (63.0%)
Overweight	1 (0.9%)
Mother’s education level, n (%)	
Illiterate & primary level	49 (45.4%)
Secondary level	45 (41.7%)
Graduation and post-graduation level	14 (13.0%)
Socioeconomic status, n (%)	
Middle class	43 (39.8%)
Lower class	51 (47.2%)
Upper class	14 (13.0%)
Efficacy, n (%)	
Yes	96 (88.9%)
No	12 (11.1%)

A total of 96 patients (88.9%) demonstrated efficacy, indicating that their hemoglobin levels were normalized after treatment. Meanwhile, 12 patients (11.1%) did not achieve normal hemoglobin levels, which may be attributed to confounding factors such as medication compliance, nutritional status of the children, maternal education level, socioeconomic status, and other illnesses. Independent samples T-tests were conducted to compare the post-treatment hemoglobin and post-treatment ferritin level between the two groups of Efficacy (Yes, No). The Mann-Whitney U test showed that these results were statistically significant. For post-treatment Hb level: U = 0.00, mean difference = 2.20, p <0.001; and for post-treatment ferritin level: U = 175, mean difference = 2.00, p <0.001. The significant p-values allowed us to reject the null hypothesis of no difference between groups. This indicates that ferrous sulfate treatment led to a statistically significant improvement in hemoglobin levels over 3 months.

The relationship between pretreatment hemoglobin (Hb) levels and weight was explored using Pearson’s correlation matrix, which indicated a weak positive correlation between pre-treatment Hb levels and weight (Pearson’s r = 0.250, p = 0.009).

## Discussion

This study aimed to assess the efficacy of ferrous sulfate in improving iron deficiency anemia (IDA) in pediatric patients. The results showed a net increase of 3.11 g/dL in hemoglobin levels compared to the pretreatment levels. The mean post-treatment ferritin was 17.7 ± 3.34 (range 12 to 28) ng/mL, reflecting a net increase of 5.9 ng/mL compared to pretreatment levels. These findings suggest that ferrous sulfate is an effective therapeutic agent for iron deficiency anemia in children.

The observed increase in hemoglobin and ferritin levels aligns with previous studies in the literature that reported similar ranges of Hb improvement following oral iron supplementation. For instance, one study reported that ferrous sulfate increased hemoglobin by 1.0 g/dL more than the iron polysaccharide complex. The median serum ferritin level increased from 3.0 to 15.6 ng/mL (ferrous sulfate) [11]. Multiple studies have indicated that both ferrous sulfate and iron polymaltose complex are similarly effective in increasing hemoglobin levels in children with iron deficiency anemia. They have reported equal efficacy of both supplements [7, 12]. 

However, some studies suggest that ferrous bisglycinate is better than ferrous sulfate in increasing mean hemoglobin levels. For instance, a study showed that after 12 weeks of treatment, the mean increase in hemoglobin was 1.8 ±1.59 g/dL in the ferrous sulphate group as compared to 2.5 ±1.31 g/dL in the ferrous bisglycinate group, showing a higher level of rise with ferrous bisglycinate than with ferrous sulphate, p = 0.0033 [13].

Our study found a weak correlation between iron deficiency anemia and underweight children. Similar results are cited by many authors, including one study that reported that “the likelihood of contracting anemia was significantly higher among underweight children compared with those who were not underweight (OR: 1.58; 95% CI: (1.11, 2.26))” [14]. However, many studies have reported a correlation between iron deficiency anemia and overweight and obese patients, and reported a high incidence of IDA in obese patients. For example, in one systematic review, the authors reported that “children living with obesity had significantly lower levels of hemoglobin, iron, % transferrin saturation, and higher levels of ferritin and hepcidin than children without obesity” [15]. Similarly, in one study, the authors reported that "iron deficiency anemia is common in overweight and obese children. A significantly greater proportion of obese than normal-weight children have IDA. Insufficient dietary intake of iron, whether absolute or relative to body mass, and increased iron needs may be a result of unbalanced nutrition or repeated short-term restrictive diets" [16].

The strengths of the present study include its clear focus on pediatric patients and its ability to address a critical age group where iron deficiency has substantial developmental consequences. Additionally, measuring both Hb and ferritin levels provided comprehensive insights into immediate and storage-related iron replenishment.

Despite its strengths, this study has several limitations. The lack of a control group restricts the ability to attribute improvements solely to ferrous sulfate. Additionally, the short follow-up period prevented the evaluation of long-term efficacy and recurrence rates. Another limitation is the absence of the assessment of adherence and potential side effects, which significantly influence the outcomes in clinical practice. Furthermore, it was conducted at a single tertiary care children’s hospital, where, in addition, a disproportionately large number of lower socioeconomic status patients present for treatment. 

Future research should address these gaps by incorporating randomized controlled designs, involving multiple centers, extending follow-up periods, and measuring long-term outcomes with adverse effects of treatment.

## Conclusions

Iron deficiency anemia is a widespread global health problem affecting children and has significant implications on growth, cognitive development, and general welfare. This study evaluated the efficacy of ferrous sulfate in the management of iron deficiency anemia in children. Key findings indicate that ferrous sulfate is highly effective in increasing mean hemoglobin and ferritin levels in children with iron deficiency anemia for 03 months. This treatment led to improvements in both clinical and laboratory parameters. These results support the use of ferrous sulfate as an effective therapeutic intervention for IDA in children. However, this study was limited by the lack of a comparison or control group, small sample size, short follow-up period, and being conducted at a single center. Randomized controlled trials involving multiple centers, comparing the efficacy of ferrous sulfate with other iron supplementation or a control group with larger sample sizes and longer follow-up periods, are recommended for future research.

## Figures and Tables

**Figure 1:**
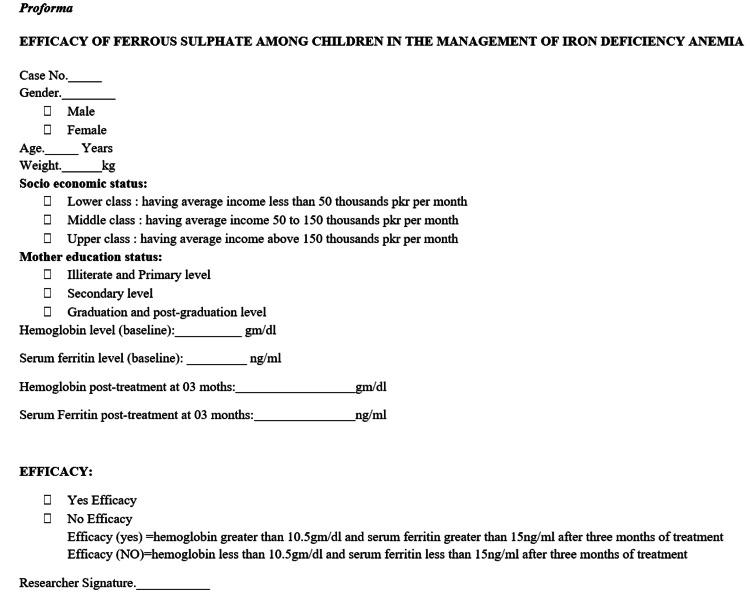
Proforma for data collection
